# Characterizing the emergence of amyloid and tau burden in Down syndrome

**DOI:** 10.1002/alz.13444

**Published:** 2023-08-29

**Authors:** Matthew D. Zammit, Tobey J. Betthauser, Andrew K. McVea, Charles M. Laymon, Dana L. Tudorascu, Sterling C. Johnson, Sigan L. Hartley, Alexander K. Converse, Davneet S. Minhas, Shahid H. Zaman, Beau M. Ances, Charles K. Stone, Chester A. Mathis, Annie D. Cohen, William E. Klunk, Benjamin L. Handen, Bradley T. Christian, the Alzheimer's Biomarker Consortium – Down Syndrome

**Affiliations:** ^1^ University of Wisconsin‐Madison Waisman Center Madison Wisconsin USA; ^2^ University of Wisconsin‐Madison Alzheimer's Disease Research Center Madison Wisconsin USA; ^3^ Department of Medicine University of Wisconsin‐Madison Madison Wisconsin USA; ^4^ Department of Radiology University of Pittsburgh Pittsburgh Pennsylvania USA; ^5^ Department of Psychiatry University of Pittsburgh Pittsburgh Pennsylvania USA; ^6^ Cambridge Intellectual Disability Research Group University of Cambridge Cambridge UK; ^7^ Department of Neurology Washington University in St. Louis St. Louis Missouri USA; ^8^ Department of Medical Physics University of Wisconsin‐Madison Madison Wisconsin USA

**Keywords:** amyloid, amyloid chronicity, Down syndrome, longitudinal, PET, Tau, trajectory modeling

## Abstract

**INTRODUCTION:**

Almost all individuals with Down syndrome (DS) will develop neuropathological features of Alzheimer's disease (AD). Understanding AD biomarker trajectories is necessary for DS‐specific clinical interventions and interpretation of drug‐related changes in the disease trajectory.

**METHODS:**

A total of 177 adults with DS from the Alzheimer's Biomarker Consortium‐Down Syndrome (ABC‐DS) underwent positron emission tomography (PET) and MR imaging. Amyloid‐beta (Aβ) trajectories were modeled to provide individual‐level estimates of Aβ‐positive (A+) chronicity, which were compared against longitudinal tau change.

**RESULTS:**

Elevated tau was observed in all NFT regions following A+ and longitudinal tau increased with respect to A+ chronicity. Tau increases in NFT regions I‐III was observed 0–2.5 years following A+. Nearly all A+ individuals had tau increases in the medial temporal lobe.

**DISCUSSION:**

These findings highlight the rapid accumulation of amyloid and early onset of tau relative to amyloid in DS and provide a strategy for temporally characterizing AD neuropathology progression that is specific to the DS population and independent of chronological age.

**Highlights:**

- Longitudinal amyloid trajectories reveal rapid Aβ accumulation in Down syndrome
- NFT stage tau was strongly associated with A+ chronicity
- Early longitudinal tau increases were observed 2.5–5 years after reaching A+

## 1 BACKGROUND

The Down syndrome (DS) population is genetically predisposed to develop Alzheimer's disease (AD) in part due to the triplication of chromosome 21 and the resulting increase in amyloid precursor protein (APP).^1^, ^2^ Increased dementia prevalence is observed after age 50 in DS^3^ with an average onset age of circa 55.^4^, ^5^ Amyloid‐beta (Aβ) plaque density has been characterized using [^11^C] Pittsburgh Compound‐B (PiB) positron emission tomography (PET) imaging. The spatial progression of Aβ in DS closely resembles that of neurotypical populations at risk for sporadic late‐onset AD.^6^, ^7^, ^8^, ^9^, ^10^, ^11^, ^12^, ^13^, ^14^, ^15^, ^16^ In addition, striatum‐first Aβ PET signal emerges in DS similar to the observations in autosomal‐dominant AD (ADAD) and APP duplication.^17^, ^18^, ^19^, ^20^ Our previous longitudinal studies in DS revealed Aβ increased by 3%–4% annually for individuals with high Aβ burden, albeit with a large distribution in the age of Aβ‐positive (A+) onset ranging from ∼30 to 60 years.^21^, ^22^, ^23^, ^24^

In agreement with late‐onset AD, the spatial progression of neurofibrillary tau tangles and paired helical filament tau in DS when measured with [^18^F]AV‐1451(flortaucipir)^25^ PET closely follows the hierarchical staging pattern outlined by Braak and Braak.^26^, ^27^, ^28^, ^29^ Comparing Aβ and tau in DS revealed higher tau deposition in limbic and neocortical regions with higher Aβ burden^28^ and significant tau PET signal was observed in Braak/NFT stage regions I‐III for individuals at the threshold of A+.^29^ Annual rates of Aβ change in these individuals were on the order of 1%–2%, suggesting a short latency period exists between emergences of these biomarkers. In neurotypical populations, tau has been associated with both Aβ and cognitive decline,^30^ and the associations between tau and cognition have been observed with a stronger effect as compared to Aβ and cognition.^31^ Early studies of tau PET in DS revealed that higher tau burden corresponded with mild cognitive impairment^16^ and that tau PET was significantly associated with cortical atrophy^32^ and accelerated longitudinal decline in cognition.^33^ For non‐demented individuals with DS, episodic memory decline was most associated with high tau burden, indicating that evaluating these biomarkers in tandem is a sensitive measure to detect early AD progression.^34^

Of interest is the use of longitudinal data to characterize biomarker change throughout the time course of AD. One method applied group‐based trajectory modeling (GBTM) to longitudinal Aβ PET data in cases of sporadic AD to model Aβ change with respect to an A+ threshold.^35^ The output of the model provided estimates of A+ chronicity, or the length of time an individual is estimated to have been A+, which was strongly associated with tau burden and cognitive decline.^35^ A study comparing GBTM to two new autonomous longitudinal modeling methods (Ordinary differential equation – Gaussian Process [ODE‐GP]; and sampled iterative local approximation [SILA]) in multiple sporadic AD cohorts validated these methods for modeling Aβ accumulation over time, predicting antecedent Aβ levels from a single scan, and estimating A+ onset age.^36^ This study also found that Aβ accumulation rates were not impacted by age, sex, or apolipoprotein E (*APOE*) genotype, and demonstrated strong associations between *APOE* genotype (but not sex) and estimated A+ onset age.^36^

Characterizing the estimated years to symptom onset (EYO) has been carried out in ADAD by subtracting an individual's age from the expected age of clinical symptom onset.^37^ Studies have taken a similar approach to define “EYO” in DS,^5^, ^38^, ^39^ and comparing these groups revealed an earlier onset of Aβ PET signal in ADAD.^38^ These studies used an estimated age of dementia onset of ∼55 years for DS, but previous findings identified a wide age range of dementia onset spanning 25+ years in DS,^5^, ^40^, ^41^, ^42^, ^43^ adding uncertainty to the EYO measure. While the expected age of symptom onset in ADAD is consistent based on specific mutations,^44^ a recent study has demonstrated that variability in predicting symptom onset is similar between DS and ADAD,^5^ suggesting that the EYO measure can be further refined. In the current work, our goal was to minimize the uncertainty in the measurement of EYO in DS by directly linking the time estimates and tau burden to well‐characterized Aβ PET data using the SILA algorithm^36^ across participants from the Alzheimer's Biomarker Consortium‐Down Syndrome (ABC‐DS) study.^45^

## 2 METHODS

### 2.1 Participants

The current sample included 177 adults with DS (89 male/88 female; mean age = 39.2 [SD = 8.50] years) recruited by the University of Wisconsin‐Madison, University of Pittsburgh, and University of Cambridge sites of the ABC‐DS study. Institutional Review Board approval and informed consent were obtained during enrollment into the study by the participant or legally designated caregiver according to the Declaration of Helsinki. Inclusion criteria required being aged ≥ 25 years and having a non‐verbal mental age of at least 3 years, based upon the Stanford‐Binet Intellectual Scales, fifth edition.^46^ Genetic testing was performed to confirm DS (full trisomy 21, mosaicism, or partial translocation). Exclusion criteria included having an unstable psychiatric or medical condition (e.g., untreated) that impaired cognitive functioning or was contraindicative of brain imaging scans (e.g., metallic implants). Participant demographics are outlined in Table 1.

**TABLE 1 alz13444-tbl-0001:** Demographics for the participants with Down syndrome.

Total Number of participants	177
Participants with mosaicism (%)	4 (2.3)
Participants with partial translocation (%)	9 (5)
Sex (M/F)	89/88
Chronological age (years)	39.2 (8.50)
*APOE* ε4 carriers (%)	35 (19)
Participants with 1 Aβ scan (%)	58 (33)
Participants with 2 Aβ scans (%)	64 (36)
Participants with 3 Aβ scans (%)	15 (8)
Participants with 4 Aβ scans (%)	30 (17)
Participants with 5 Aβ scans (%)	10 (6)
Participants with 1 tau scan (%)	75 (45)
Participants with 2 tau scans (%)	92 (55)

### 2.2 Imaging

T1‐weighted magnetic resonance imaging (MRI) scans were acquired on a GE Signa 750 (Wisconsin), Siemens Trio or Prisma (Pittsburgh), and GE SIGNA PET/MR (Cambridge). The MRI images were processed using FreeSurfer v5.3.0 for region of interest (ROI) definition. Positron emission tomography (PET) scans were performed on a Siemens ECAT HR+ scanner (Wisconsin/Pittsburgh), Siemens four‐ring Biograph mCT (Pittsburgh), and GE Signa PET/MR (Cambridge). A target dose of 15 mCi of [C‐11]Pittsburgh Compound‐B (PiB) was injected intravenously, and PET scans were used to measure Aβ acquired 50–70 min post‐injection (four 5‐min frames). Several hrs following completion of the PiB scan, a target dose of 10 mCi of [F‐18]AV‐1451 was injected intravenously, and PET scans were used to measure neurofibrillary tau acquired 80–100 min post‐injection (four 5‐min frames). Using the Statistical Parametric Mapping 12 software (SPM12), PET frames were re‐aligned to correct motion and averaged to form a 3D image. All participants were scheduled to undergo PiB imaging every 2–3 years; 58 had one scan, 64 had two, 15 had three, 30 had four, and the remaining 10 had five scans. To date, only 167 individuals completed the AV‐1451 imaging; 75 had a single scan, and the remaining 92 had two scans. All PiB and AV‐1451 scans were performed on the same day.

**RESEARCH IN CONTEXT**

1. **Systematic review**: Positron emission tomography (PET) imaging studies in individuals with Down syndrome (DS) have revealed early and aggressive accumulation of amyloid‐beta (Aβ). At the cross‐sectional level, early tau deposition has been observed in individuals shortly after reaching Aβ‐positivity (A+). To date, a longitudinal tau PET analysis has yet to be performed in DS.
2. **Interpretation**: Our findings present the first longitudinal evaluation of tau change in AD with DS. By relating tau change to duration of A+ (A+ chronicity), it was observed that tau increases emerged within 2.5–5 years following A+.
3. **Future directions**: A+ chronicity is a powerful tool that can accurately characterize the progression of AD biomarker change in DS. A direct comparison of tau change in DS and late‐onset AD relative to A+ chronicity should be performed to better define potential AD treatments for individuals with DS.

### 2.3 Aβ PET quantification

PiB PET images were spatially normalized to the Montreal Neurological Institute 152 space (MNI152) via a DS‐specific PET template for PiB as previously described.^11^ Using gray matter cerebellum as a reference tissue, standardized uptake value ratio (SUVR) images were generated through voxel normalization of summed PET images. Global Aβ burden was determined using the amyloid load metric (Aβ_L_),^47^ derived from all Aβ‐carrying voxels in an SUVR image by linear least squares fitting of DS‐specific template images representing Aβ carrying capacity and PiB nonspecific binding, as described previously.^23^ Aβ_L_ was chosen as the primary outcome measure for Aβ burden as it suppresses the nonspecific binding component of voxels in the image and shows increased power to detect early changes in Aβ compared to SUVR/Centiloids (CL).^47^ Our previous work comparing both Aβ_L_ and CL in DS identified the following linear relation between metrics^29^:[Table alz13444-tbl-0001]

(1) $$Equivalent\;CL\;=\;2.27*A\beta_{L}-12.1$$

For all analyses, the Aβ burden will be presented using both Aβ_L_ and its CL equivalent. Previous longitudinal PiB PET analysis in DS identified an early amyloid positive (A+) cutoff of 13.3 Aβ_L_ corresponding to 18.0 CL.^24^

### 2.4 Tau PET quantification

Baseline and follow‐up AV‐1451 PET images were registered to the baseline T1w‐MRI images for each participant, and SUVR images were generated by voxel normalization to gray matter cerebellum 80–100 min post‐injection. No erosion or elimination of regions of focal uptake of AV‐1451 was performed on the cerebellar gray matter ROI used for signal normalization. Using FreeSurfer v5.3.0, ROI masks from multiple brain regions were delineated from the T1w‐MRI. The ROI masks were then combined to create ROIs consistent with regions used for NFT stages I‐VI as described previously,^48^ which were used to extract NFT stage SUVRs. For all longitudinal AV‐1451 scans, the NFT stage ROI masks from the baseline MRI time point were used for SUVR extraction.

### 2.5 Aβ trajectory modeling

Longitudinal Aβ trajectories were modeled using a sampled iterative local approximation (SILA) algorithm (https://github.com/Betthauser‐Neuro‐Lab/SILA‐AD‐Biomarker) as described previously.^36^ Briefly, the relationship between Aβ rate of change and Aβ burden was established by discrete sampling of the Aβ versus chronological age data for participants with ≥2 PiB PET scans. A non‐parametric fit of Aβ burden versus A+ chronicity was generated by integrating the Aβ_L_ rate versus Aβ_L_ data using Euler's method, with the initial condition that A+ chronicity of 0 years corresponds to the A+ cutoff of 13.3 Aβ_L_ (18.0 CL). Numerical integration was performed with a 0.25‐year step size across 200 iterations (corresponding to 50 years of disease time). The algorithm was restricted to integrating individual‐level data for participants with two or more time points and terminated if the Aβ slope was negative or if the maximum number of iterations was reached. For participants with only a single Aβ scan, their A+ chronicity was numerically estimated by solving the fit of the nonparametric Aβ versus time curve at their given value of Aβ burden. For all other participants with longitudinal data, their Aβ trajectories were aligned to the curve based on the scan at which they became A+. Thus, the A+ chronicity estimate for these individuals was centered based on their true A+ time point during the study period. For individuals that did not reach the A+ threshold, their most recent scan was aligned to the Aβ versus time curve. If an individual's Aβ burden did not fall within the modeled range, their A+ chronicity was truncated to the earliest modeled value on the Aβ versus time curve. For each participant, an estimated age of A+ onset was determined by subtracting the estimated A+ chronicity at their reference scan from their chronological age at that scan.

### 2.6 Statistical analyses

Rates of Aβ accumulation are presented as mean with standard deviation (SD). Comparisons of estimated A+ onset age between groups were performed using the Student's t‐tests. Associations between NFT stage tau, chronological age, Aβ burden, and A+ chronicity were evaluated using Pearson's correlation coefficients with 95% confidence intervals (CIs). *Effect sizes*: Significant increases in tau change between baseline and follow‐up scans were evaluated using Cohen's d effect sizes^49^ with 95% CIs.

## 3 RESULTS

### 3.1 Modeling Aβ trajectories identifies A+ chronicity time and estimated A+ onset age in DS

**FIGURE 1:**
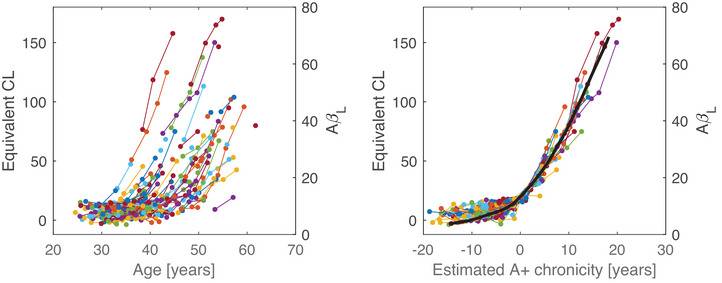
Longitudinal amyloid‐beta (Aβ) burden (represented as amyloid load [Aβ_L_] and equivalent centiloids [CL]) with respect to age (left). Sampled iterative local approximation (SILA) modeled fit of Aβ burden with respect to estimated Aβ‐positive (A+) chronicity (right). Single points represent participants with only one Aβ scan, while points connected by lines represent participants with longitudinal data. Each color represents a single individual.

**FIGURE 2:**
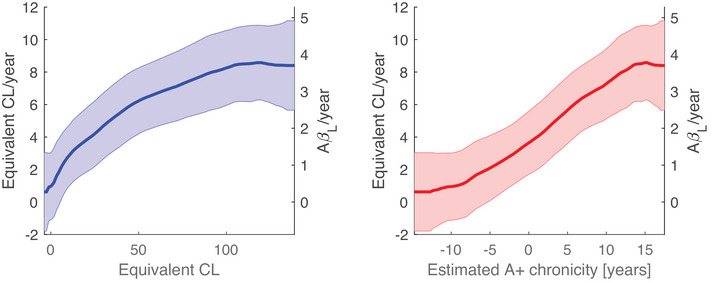
Longitudinal rate of equivalent centiloids (CL) and amyloid load (Aβ_L_) (presented as change/year) with respect to amyloid‐beta (Aβ) burden (left) and Aβ‐positive (A+) chronicity (right). Shaded regions represent the standard deviation.

Using the SILA algorithm, a longitudinal Aβ trajectory curve was created to represent Aβ burden across a ∼30‐year span (Figure 1), consistent with the estimated disease timeline in neurotypical populations as described elsewhere.^50^, ^51^ Modeled Aβ_L_ and equivalent CL data points for each A+ chronicity time are displayed in Table S1. Aligning each participant's observed longitudinal data to this trajectory based on their first A+ timepoint suggested reasonable model fits for later time points after an A+ chronicity time of 0 years (13.3 Aβ_L_ or 18.0 CL). Figure 2 displays the annual rates of Aβ change with respect to both Aβ burden and A+ chronicity as modeled by the SILA algorithm. Here, we observed a continuous increase in the rate of Aβ accumulation as Aβ burden increased, with a plateau in the rate of change observed as 8.45 (2.42) CL/year or 3.73 (1.07) Aβ_L_/year. At an A+ chronicity time of 0 years, the mean rate of Aβ increase was 3.65 (1.94) CL/year or 1.60 (0.86) Aβ_L_/year. Across this cohort, the estimated age of A+ onset for *APOE* ε4 carriers (*N* = 35) was 40.1 [38.5–41.7] years, while non‐carriers reached A+ at 41.6 [40.5, 42.6] years. While not statistically significant (*p*> 0.05 from Student's *t*‐test), the earlier onset of A+ in *APOE* ε4 carriers in this cohort is in agreement with other studies of DS.^52^

### 3.2 NFT stage tau is associated with chronological age

**FIGURE 3:**
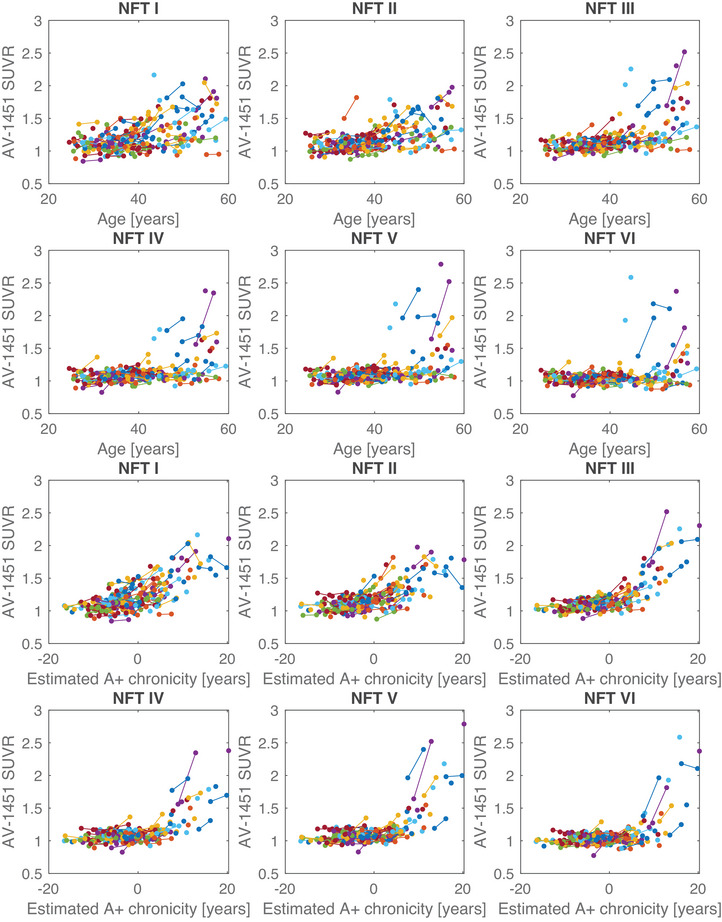
Tau burden (presented as AV‐1451 standardized uptake value ratios [SUVR]) within each neurofibrillary tau (NFT) stage with respect to chronological age and Aβ‐positive (A+) chronicity.

Increases in tau were observed across all NFT stage regions with respect to chronological age (Figure 3, top two rows). Pearson correlation analysis (presented as Pearson's *r* with 95% CIs) revealed significant associations (*p* < 0.05) between tau and chronological age across all NFT stage regions: NFT I *r* = 0.48 [0.37, 0.59], NFT II *r* = 0.49 [0.38, 0.60], NFT III *r* = 0.52 [0.41, 0.62], NFT IV *r* = 0.43 [0.30, 0.54], NFT V *r* = 0.42 [0.29, 0.53], NFT VI *r* = 0.28 [0.14, 0.41].

### 3.3 NFT stage tau is highly associated with A+ chronicity

Nearly all individuals with DS revealed increased tau burden in NFT regions I‐IV following A+ onset (Figure 3, bottom two rows). Pearson analysis showed statistically significant associations (*p*< 0.05) between Aβ and tau across all six NFT stage regions as follows: NFT I *r* = 0.71 [0.63, 0.77], NFT II *r* = 0.68 [0.60, 0.75], NFT III *r* = 0.81 [0.75, 0.85], NFT IV *r* = 0.66 [0.57, 0.77], NFT V *r* = 0.67 [0.59, 0.75], NFT VI *r* = 0.63 [0.53, 0.71]. Pearson's statistics reveal that NFT stage tau has a higher association with Aβ than with chronological age. From Pearson analysis, significant associations between A+ chronicity and tau were observed across all NFT stage regions: NFT I *r* = 0.69 [0.60, 0.76], NFT II *r* = 0.65 [0.56, 0.73], NFT III *r* = 0.69 [0.61, 0.76], NFT IV *r* = 0.57 [0.46, 0.66], NFT V *r* = 0.57 [0.46, 0.66], NFT VI *r* = 0.50 [0.38, 0.60] (Figure 3). Similar to Aβ_L_, Pearson analysis shows that a higher association was observed between A+ chronicity and NFT stage tau burden compared to chronological age. As A+ chronicity is dependent on the Aβ burden, correlations between Aβ_L_ and tau, and between A+ chronicity and tau were similar and not statistically different.

### 3.4 Longitudinal tau increases rapidly emerge following A+

**FIGURE 4:**
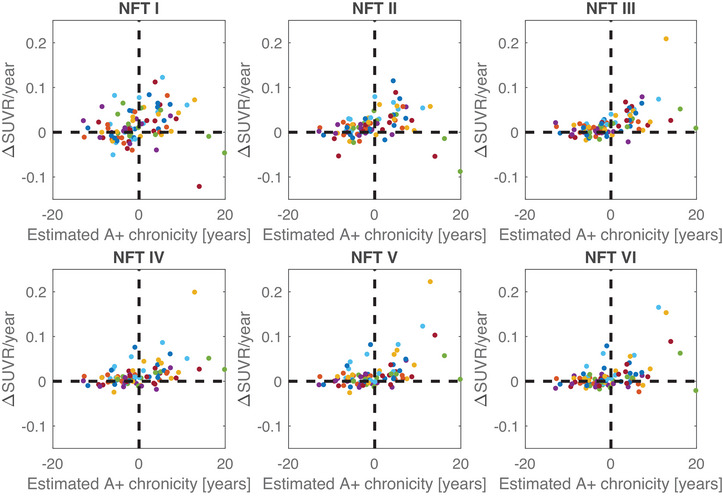
Longitudinal tau change (presented as standardized uptake value ratio change per year [SUVR/year]) across each neurofibrillary tau (NFT) stage region with respect to Aβ‐positive (A+) chronicity. Dashed lines represent zero tau change (horizontal) and an A+ chronicity of 0 years (vertical). Each colored point represents a single individual with Down syndrome (DS).

**FIGURE 5:**
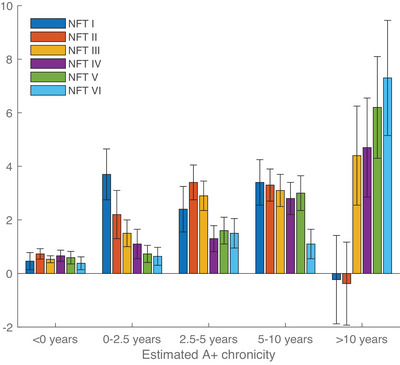
Annualized % change in tau burden within each neurofibrillary tau (NFT) stage region categorized by bin of Aβ‐positive (A+) chronicity. Error bars represent the 95% confidence interval.

To characterize the emergence of tau relative to amyloid, longitudinal tau change was measured across all six NFT stage regions and categorized into different bins of A+ chronicity encompassing the early and late stages of Aβ accumulation. A+ chronicity bins were selected to have a similar number of individuals in each bin for A+ chronicity ≥ 0 years. For the participants with longitudinal tau data available, the rate of tau change in SUVR/year was compared against A+ chronicity (Figure 4). Table 2 displays the annualized percent change in tau for each NFT region across the different bins of A+ chronicity, while Table 3 displays the Cohen's d effect size of annualized longitudinal change between the baseline and follow‐up scans. For A+ chronicity < 0 years, small effect sizes (Cohen's d < 0.5) were observed between baseline and follow‐up tau burden. Between A+ chronicity of 0–2.5 years, NFT regions I and III displayed large effect sizes (Cohen's d > 0.8), while NFT regions II and IV displayed medium effect sizes (Cohen's d > 0.5). The lower effect size in NFT region II compared to NFT regions I and III is likely an effect of both choroid plexus signal spillover and hippocampal atrophy. For an estimated A+ chronicity of 2.5–5 years, NFT regions I–II and IV–VI display medium effect sizes, while NFT region III displays a large effect size with respect to tau increase. Longitudinal tau increase is represented by medium to large effect sizes for all NFT stage regions after an A+ chronicity of 5 years, and beyond 10 years, no change is observed in NFT regions I‐II. When visualizing the annualized percent change in tau burden relative to A+ chronicity (Figure 5), tau burden in NFT regions I‐III has the greatest magnitude of increase with lower A+ chronicity. As A+ chronicity increases, tau change in NFT regions I‐II becomes static while NFT regions III‐VI continue to rapidly increase. These findings reveal that the rates of tau change in DS are consistent with the proposed spatiotemporal tau deposition patterns outlined by Braak and Braak, and that the A+ chronicity measure can provide a timeline of tau progression throughout the development of AD.

**TABLE 2:** Longitudinal rates of tau change (annualized % change presented as mean [95% CI]) across each NFT stage region of tau pathology with respect to A+ chronicity (years).

A+ chronicity	N	Aβ burden	NFT I	NFT II	NFT III	NFT IV	NFT V	NFT VI
<0 years	56	7.61 CL	0.46[0.14, 0.78]	0.73[0.54, 0.93]	0.53[0.40, 0.66]	0.66[0.45, 0.87]	0.59[0.36, 0.83]	0.38[0.14, 0.62]
0–2.5 years	8	22.2 CL	3.7[2.8, 4.7]	2.2[1.3, 3.1]	1.5[1.0, 2.0]	1.1[0.55, 1.7]	0.73[0.41, 1.1]	0.64[0.31, 0.98]
2.5–5 years	12	34.7 CL	2.4[1.6, 3.3]	3.4[2.8, 4.1]	2.9[2.4, 3.5]	1.3[0.82, 1.8]	1.6[1.1, 2.1]	1.5[0.95, 2.1]
5–10 years	11	55.3 CL	3.4[2.6, 4.3]	3.3[2.7, 3.9]	3.1[2.5, 3.7]	2.8[2.2, 3.4]	3.0[2.4, 3.7]	1.1[0.55, 1.7]
>10 years	5	107 CL	−0.23[−1.9, 1.4]	−0.38[−1.9, 1.2]	4.4[2.6, 6.3]	4.7[2.9, 6.6]	6.2[4.3, 8.1]	7.3[5.2, 9.5]

*Note*: Early tau increases were observed within 2.5 years following A+ onset.

**TABLE 3:** Effect size (Cohen's d [95% CI]) between baseline and follow‐up AV‐1451 SUVR for each NFT stage region.

A+ chronicity	NFT I	NFT II	NFT III	NFT IV	NFT V	NFT VI
<0 years	0.12[−0.25, 0.49]	0.29[−0.083, 0.66]	0.3[−0.073, 0.67]	0.27[−0.10, 0.64]	0.24[−0.13, 0.61]	0.14[−0.23, 0.51]
0–2.5 years	1.0[−0.062, 2.0]	0.63[−0.39, 1.6]	0.84[−0.20, 1.9]	0.59[−0.42, 1.6]	0.41[−0.59, 1.4]	0.37[−0.63, 1.4]
2.5–5 years	0.53[−0.29, 1.3]	0.69[−0.14, 1.5]	1.1[0.22, 2.0]	0.61[−0.22, 1.4]	0.78[−0.060, 1.6]	0.64[−0.19, 1.5]
5–10 years	0.75[−0.12, 1.6]	1.0[0.098, 1.9]	1.3[0.27, 2.1]	1.2[0.27, 2.1]	1.2[0.27, 2.1]	0.43[−0.42, 1.3]
>10 years	−0.017[−1.3, 1.2]	−0.092[−1.3, 1.2]	1.0[−0.36, 2.3]	0.81[−0.51, 2.1]	0.88[−0.45, 2.2]	0.77[−0.55, 2.0]

*Note*: For an A+ chronicity of 0–2.5 years, medium to large effect sizes were observed for NFT regions I‐III. For an A+ chronicity of 2.5–5 years, medium to large effect sizes were observed in all NFT stage regions.

## 4 DISCUSSION

We present the first longitudinal tau PET analysis in the DS population. Using the measure of A+ chronicity, a timeline of the progression of tau through the conventional NFT stages was established for DS, revealing early and rapid tau change with A+ onset. When characterizing the progression of AD, it is important to understand the time course of biomarkers such as Aβ and neurofibrillary tau to inform diagnosis and relevant treatment strategies. Relying on age alone to estimate the years to symptom onset (EYO) is challenging due to the heterogeneity in the expected age of onset, which varies between sporadic and familial AD types^44^, ^53^ and also varies in the DS population.^4^, ^5^ Given that the DS population is genetically predisposed to AD, their AD trajectories are often directly compared to autosomal‐dominant forms of AD.^38^, ^54^ However, prediction of EYO in ADAD is more reliable than in DS, where a wide distribution in age of onset has been observed.^5^, ^40^, ^41^, ^42^, ^43^ This may be because EYO in ADAD is continuously refined based on individuals with close genetic relationships in a given family; thus, progressively narrowing the range of other genetic variability in the group that determines the EYO value. Measuring cognitive decline in individuals with DS can also be challenging due to their lower and varied baseline intellectual functioning level compared to neurotypical populations,^55^, ^56^, ^57^ adding further uncertainty to age‐based EYO classification. However, recent studies identified that measuring cognitive decline in the domain of episodic memory is a powerful tool to study AD progression in DS, as significant changes in episodic memory scores were observed in association with higher tau burden^34^ and PET measures of neurodegeneration.^58^ Here, we present findings that resolve some of the challenges associated with age‐based measures of EYO by directly linking the time course of AD progression to well‐characterized biomarker measures. By using A+ chronicity, or the estimated years from Aβ onset, a more disease‐specific timeline of AD progression can be achieved compared to age or other indirect measures of disease stage.

Comparing longitudinal tau PET change to the A+ chronicity metric identified a short latency period between A+ onset and early‐stage tau deposition in the DS population. For an A+ chronicity greater than 0 years, nearly all individuals with DS had elevated tau and increases in tau burden in NFT stage regions I‐VI. Significant tau increases were observed within the first 2.5 years of A+ in NFT regions I‐IV, while NFT regions V‐VI showed significant increases within 5 years of A+ onset. These findings recapitulate the Braak staging pattern of tau^27^ and provide new evidence of a spatiotemporal pattern of tau progression in DS. Many individuals displayed very early increases in the AV‐1451 signal in NFT region II near an A+ chronicity of 0 years. These early signal increases were likely attributed to signal spill‐in from off‐target choroid plexus binding, but this was not tested. With increased A+ chronicity, several individuals with DS displayed decreases in tau PET signal in the entorhinal cortex and hippocampus, and these changes were likely attributed to partial volume effects, atrophy, and ventricular enlargement observed with AD progression in DS. Similarly, the observation that medial temporal tau accumulation plateaus and begins to decline as other regions accelerate has been observed in sporadic AD.^59^ A+ chronicity has previously been associated with entorhinal tau at the cross‐sectional level in sporadic AD, where tau burden increases were estimated to occur 5–10 years following A+ onset, but with considerable inter‐individual heterogeneity in a cohort of mostly initially unimpaired, at‐risk adults.^35^ There was heterogeneity in the length of A+ chronicity required to see elevated tau burden in sporadic AD, and many individuals showed no entorhinal tau following 10 years of A+.^35^ This finding is in contrast with the observations in our DS cohort, showing an early and far more homogeneous tau increase in relation to A+ chronicity across all NFT stage regions. In sporadic AD, there is a sharp increase in tau prevalence at an Aβ threshold of 50 CL.^60^ At 50 CL in cognitively unimpaired individuals, longitudinal rates of tau increase are ∼2% per year,^61^ and annual rates of Aβ increase are ∼5 CL/year.^62^ In DS at 30 CL, we observe 2% annual increases in tau burden (Table 2) and an Aβ change rate of ∼5 CL/year. The Aβ change rate in DS increases to ∼6 CL/year at a threshold of 50 CL and continues to accelerate between 50 and 100 CL, after which the rate of increase (but not the level of Aβ) tends to plateau (Figure 2). Comparing DS and sporadic AD, we find that longitudinal tau increases emerge when Aβ rates increase at 5 CL/year, regardless of the baseline CL value. The mean rate of Aβ accumulation measured with PiB in our DS cohort was 6.9 (2.1) CL/year, compared to rates of 4–5 CL/year in sporadic AD as measured with PiB.^62^, ^63^ The accelerated deposition of Aβ observed in DS may contribute to the earlier onset of tau accumulation compared to sporadic AD. This may also suggest that, similar to ADAD,^64^ DS may have more aggressive Aβ and tau phenotypes compared to late‐onset sporadic AD. While a plateau emerged in the rate of Aβ accumulation in DS, none of the participants in our cohort displayed a plateau in Aβ burden. In sporadic AD, it has been shown that Aβ levels plateau late in the disease stage.^50^ The lack of observed Aβ plateau in our cohort may be a result of the young age of our participants (mean age = 39.2 (SD = 8.50) years), who are approximately 15 years from AD onset. Future longitudinal studies with ABC‐DS will explore the Aβ burden in older adults with DS as they age, which may provide insight on whether an Aβ plateau is reached in this population.

The measure of A+ chronicity can be a powerful tool to implement in AD treatment and prevention trials. Because A+ chronicity is modeled from well‐characterized biomarker data, it resolves many of the challenges associated with age‐based recruitment and minimizes the uncertainty associated with whether an individual is on a trajectory to develop AD. Because A+ chronicity is derived from Aβ PET, it does not provide improved predictive performance relative to a measure of Aβ_L_ or CL; however, it does help characterize the progression of Aβ with respect to disease time. In the DS population specifically, where the age of symptom onset displays a wide distribution spanning 25+ years,^5^ the metric of A+ chronicity can accurately characterize AD progression in an age‐independent manner. Due to the uniform increase in tau following A+ onset, recruitment of individuals with DS into AD‐modifying trials can be feasibly performed based on the results of a single Aβ PET scan rather than by age alone. For the purposes of this work, the SILA model was trained using longitudinal data and then applied at the individual scan level to determine the time estimates. Thus, the predictive value of the A+ chronicity measure works equally for individuals with only a single PET scan and those with longitudinal time points. A+ chronicity is not limited to the prediction of tau, as it has been shown to be an accurate predictor of cognitive decline with AD.^35^, ^36^ Furthermore, trajectory modeling strategies can be applied to longitudinal data of other AD biomarkers such as tau PET, plasma or cerebrospinal fluid measures of Aβ/tau, and cognitive data to further characterize the natural history of AD progression. For the DS population, work is ongoing to compare A+ chronicity with both cognitive and fluid‐based biomarker measures. Future work will implement the SILA method across longitudinal tau PET data and characterize tau positive (T+) duration with respect to cognitive decline.

The results presented in this work highlight the first longitudinal analysis of tau PET in the DS population and the first demonstration that individualized A+ onset ages and durations can be estimated in DS. Using A+ chronicity to characterize the natural history of AD progression identified a uniform and predictable increase in tau burden following A+. Individuals with DS uniformly displayed increases in tau in NFT regions I–IV within the first 2.5 years after A+, with NFT regions V and VI accumulating tau following 5 years of A+ chronicity. These findings highlight the early onset of tau relative to Aβ in DS compared to sporadic late‐onset AD and provide a strategy for temporally characterizing AD neuropathology progression that is specific to the DS population.

## CONFLICT OF INTEREST STATEMENT

GE Healthcare holds a license agreement with the University of Pittsburgh based on the technology described in this manuscript. Dr. Klunk is a co‐inventor of PiB and, as such, has a financial interest in this license agreement. GE Healthcare provided no grant support for this study and had no role in the design or interpretation of results or preparation of this manuscript. AVID Radiopharmaceuticals provided the precursor and reference standard to produce AV‐1451. S.C.J. has served on advisory committees to Roche, Merck, and Alzpath and has received research funding for unrelated work from Cerveau Technologies. All other authors have no conflicts of interest with this work and had full access to all the data in the study and take responsibility for the integrity of the data and the accuracy of the data analysis. The authors have no conflicts of interest. Author disclosures are available in the supporting information.

## CONSENT STATEMENT

Institutional Review Board approval and informed consent were obtained during enrollment into the study by the participant or legally designated caregiver according to the Declaration of Helsinki.

## Supporting information

Supporting Information

Supporting Information

## Appendix

*Alzheimer's Biomarker Consortium‐ Down Syndrome (ABC‐DS) Investigators:

Howard J. Aizenstein, MD PhD; Beau M. Ances, MD PhD; Howard F. Andrews, PhD; Karen Bell, MD; Rasmus M. Birn, PhD; Adam M. Brickman, PhD; Peter Bulova, MD; Amrita Cheema, PhD; Kewei Chen, PhD; Bradley T. Christian, PhD; Isabel Clare, PhD; Lorraine Clark, PhD; Ann D. Cohen, PhD; John N. Constantino, MD; Eric W. Doran, MS; Anne Fagan, PhD; Eleanor Feingold, PhD; Tatiana M. Foroud, PhD; Benjamin L. Handen, PhD; Jordan Harp, PhD; Sigan L. Hartley, PhD; Elizabeth Head, PhD; Rachel Henson, MS; Christy Hom, PhD; Lawrence Honig, MD; Milos D. Ikonomovic, MD; Sterling C Johnson, PhD; Courtney Jordan, RN; M. Ilyas Kamboh, PhD; David Keator, PhD; William E. Klunk, MD PhD; Julia K. Kofler, MD; William Charles Kreisl, MD; Sharon J. Krinsky‐McHale, PhD; Florence Lai, MD; Patrick Lao, PhD; Charles Laymon, PhD; Joseph Hyungwoo Lee, PhD; Ira T. Lott, MD; Victoria Lupson, PhD; Mark Mapstone, PhD; Chester A. Mathis, PhD; Davneet Singh Minhas, PhD; Neelesh Nadkarni, MD; Sid O'Bryant, PhD; Melissa Parisi, MD PhD; Deborah Pang, MPH; Melissa Petersen, PhD; Julie C. Price, PhD; Margaret Pulsifer, PhD; Michael S. Rafii, MD PhD, Eric Reiman, MD; Batool Rizvi, MS; Herminia Diana Rosas, MD; Laurie Ryan, PhD; Frederick Schmitt, PhD; Nicole Schupf, PhD; Wayne P. Silverman, PhD; Dana L. Tudorascu, PhD; Rameshwari Tumuluru, MD; Benjamin Tycko, MD PhD; Badri Varadarajan, PhD; Desiree A. White, PhD; Michael A. Yassa, PhD; Shahid Zaman, MD PhD; Fan Zhang, PhD.
