# Supplementary material for: Characterizing the emergence of amyloid and tau burden in Down syndrome
Source: Alzheimers Dement. 2023 Aug 29;20(1):388–98. doi: 10.1002/alz.13444 (PMC10843570; doi:10.1002/alz.13444)
Supplement: Supplementary file 1 — Supporting Information [file ALZ-20-388-s001.docx]

Supplemental Table 1. Amyloid load (Aβ_L_) and Equivalent Centiloid (CL) values from the longitudinal Aβ trajectory curve (Figure 1) with respect to A+ chronicity as modeled by the sampled iterative local approximation (SILA) algorithm.

| **A+ chronicity [years]** | **Aβ_L_** | **Equivalent CL** |
| --- | --- | --- |
| -14.0 | 3.9 | -3.2 |
| -13.0 | 4.2 | -2.6 |
| -12.0 | 4.5 | -2.0 |
| -11.0 | 4.7 | -1.3 |
| -10.0 | 5.2 | -0.3 |
| -9.0 | 5.6 | 0.7 |
| -8.0 | 6.2 | 2.0 |
| -7.0 | 6.8 | 3.3 |
| -6.0 | 7.4 | 4.6 |
| -5.0 | 7.9 | 5.7 |
| -4.0 | 8.5 | 7.1 |
| -3.0 | 9.2 | 8.9 |
| -2.0 | 10.3 | 11.2 |
| -1.0 | 11.6 | 14.2 |
| 0.0 | 13.3 | 18.0 |
| 1.0 | 15.2 | 22.3 |
| 2.0 | 17.1 | 26.7 |
| 3.0 | 19.1 | 31.3 |
| 4.0 | 21.3 | 36.3 |
| 5.0 | 23.6 | 41.6 |
| 6.0 | 26.2 | 47.3 |
| 7.0 | 29.2 | 54.1 |
| 8.0 | 32.1 | 60.8 |
| 9.0 | 35.1 | 67.6 |
| 10.0 | 38.4 | 75.1 |
| 11.0 | 42.1 | 83.4 |
| 12.0 | 45.7 | 91.7 |
| 13.0 | 49.4 | 100.0 |
| 14.0 | 53.3 | 109.0 |
| 15.0 | 57.1 | 117.6 |
| 16.0 | 60.9 | 126.1 |
| 17.0 | 64.6 | 134.5 |
| 18.0 | 68.4 | 143.2 |
